# Long-term survival with multidisciplinary treatment in metastatic sarcomatoid renal cell carcinoma: a case report and literature review

**DOI:** 10.3389/fonc.2025.1600376

**Published:** 2025-08-18

**Authors:** Xin Li, Weijia Wang, Lingjun Meng, Honglu Liang

**Affiliations:** ^1^ Department of Radiotherapy, Qilu Hospital of Shandong University Dezhou Hospital, Dezhou, China; ^2^ Department of Oncology, Qilu Hospital of Shandong University Dezhou Hospital, Dezhou, China

**Keywords:** sarcomatoid renal cell carcinoma (SRCC), renal cell carcinoma (RCC), pembrolizumab, axitinib, nephrectomy

## Abstract

**Background:**

Sarcomatoid transformation in renal cell carcinoma, termed sarcomatoid renal cell carcinoma (SRCC), is associated with aggressive behavior and an unfavorable prognosis. The presence of sarcomatoid differentiation poses a therapeutic challenge due to limited response to existing systemic therapies; however, advances in drugs with immune checkpoint inhibitor (ICI) have improved response rates.

**Case description:**

We report the case of a middle-aged woman diagnosed with multiple metastatic SRCC, treated with a combination of pembrolizumab plus axitinib as first-line therapy, transarterial chemoembolization, and nephrectomy. The patient ultimately achieved complete remission but presented with severe colitis symptoms. Following the cessation of pembrolizumab and axitinib therapy for 1 year, her colitis symptoms gradually resolved, with no evidence of recurrence or metastasis observed during that time. Her survival period has now extended beyond 2.5 years.

**Conclusion:**

Metastatic sarcomatoid renal cell carcinoma is associated with very poor prognosis, with a survival period of <1 year despite systemic therapy. In this present case, the patient achieved long-term survival following multidisciplinary treatment—a rare occurrence worthy of report.

## 1 Introduction

The sarcomatoid variant of renal cell carcinoma (RCC) is characterized by a spindle-cell phenotype and, unlike other histological RCC types, is documented to exhibit an unfavorable prognosis (1–3). Typically, sarcomatoid renal cell carcinoma (SRCC) presents as a large and invasive tumor, with metastatic potential in most cases. SRCC carries a poor prognosis, with median survival of approximately half a year, and a higher percentage of sarcomatoid components is indicative of a more adverse outcome (4). The biology of SRCC is not well understood, and its response to standard treatments is generally unsatisfactory. Many studies have highlighted that the prognosis for SRCC patients remains poor, even following treatment with tyrosine kinase inhibitors (TKI) (1).

In this report, we present our own experience with a case of multiple metastatic sarcomatoid RCC (mSRCC), in which the patient achieved long-term survival following multidisciplinary therapy. Relevant literature is also discussed.

## 2 Case report

**Figure 1:**
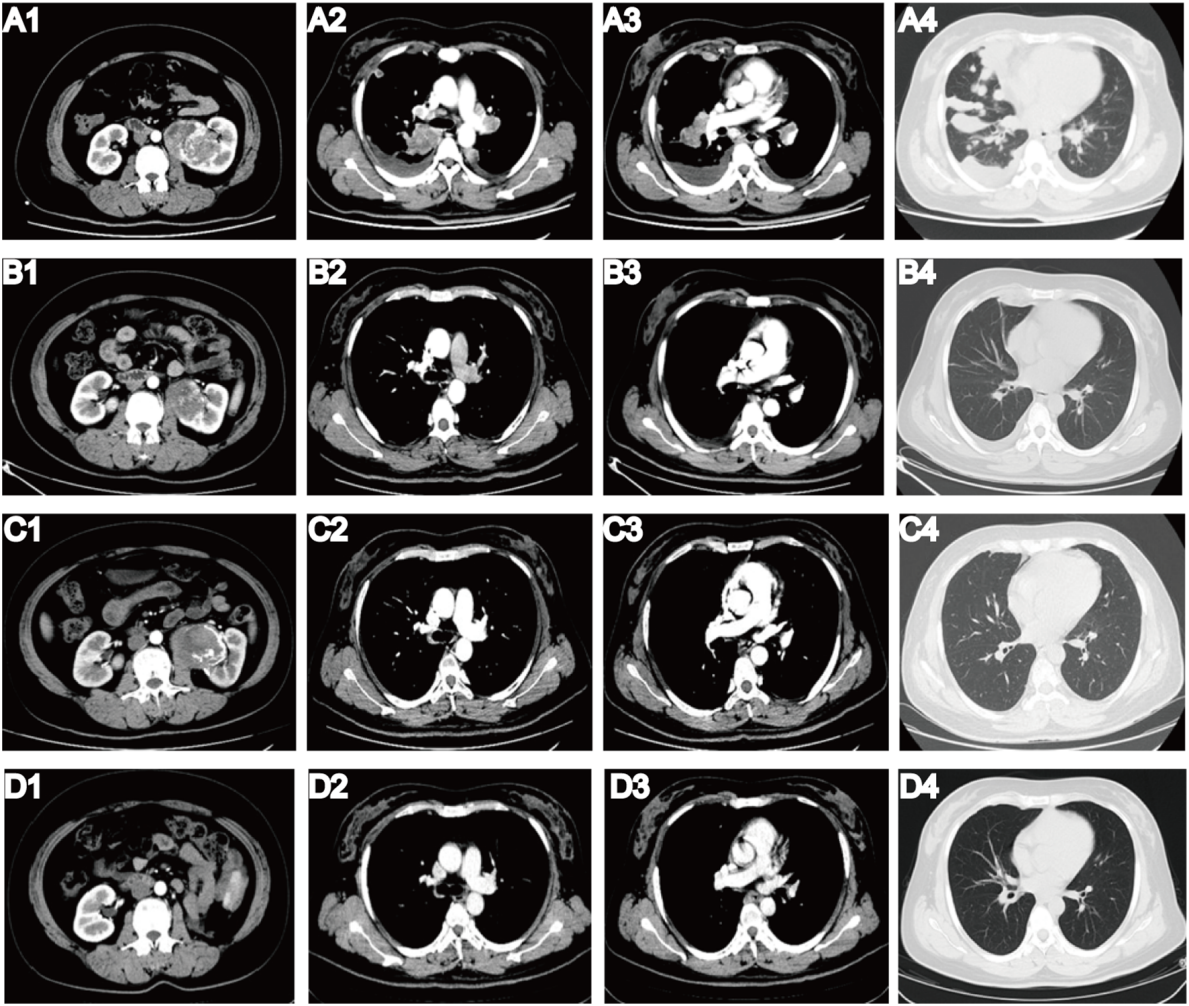
Results of enhanced CT before and after multidisciplinary treatment. **(A1)** Before multidisciplinary treatment, CT revealed a 9-cm tumor in the left kidney. **(A2–3)** CT showed bilateral pleural effusion and metastatic lesions in bilateral hilar and mediastinal lymph nodes. **(A4)** CT showed multiple metastatic lung lesions. **(B1–4)** After four cycles of pembrolizumab plus axitinib, all metastatic lesions had completely resolved, and the primary tumor volume was significantly lower than that in the prior examination. **(C1–4)** After more than 1 year of immunotherapy and transarterial chemoembolization (TACE), CT on 20 September 2023 demonstrated that the therapeutic outcome remained stable. **(D1–4)** Following multidisciplinary treatment, the patient achieved complete remission. CT in March 2025 showed no evidence of recurrence or metastasis, and follow-up is ongoing.

**Figure 2:**
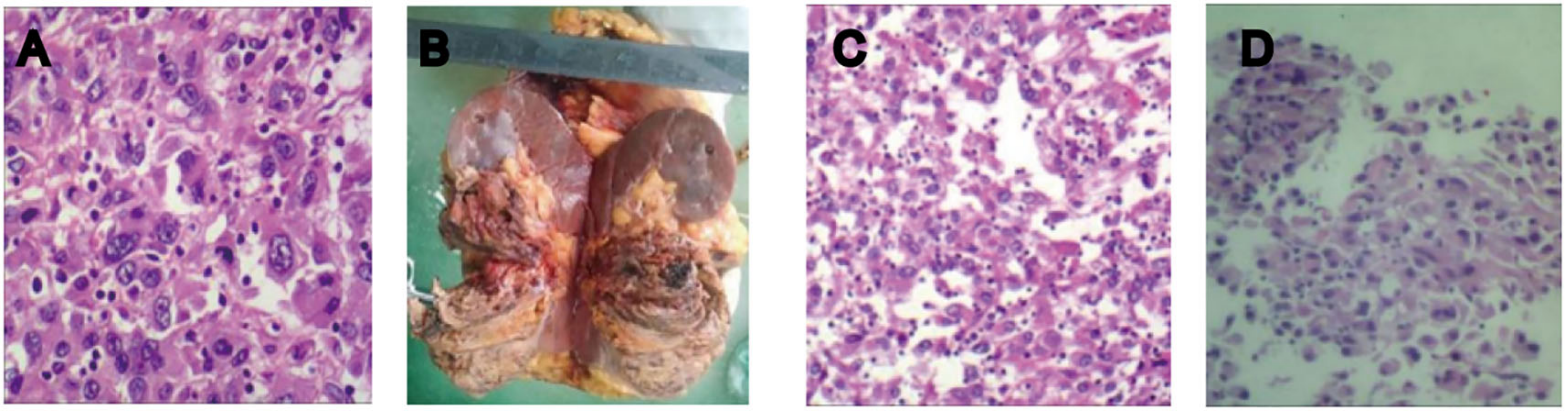
**(A)** Histopathology of the biopsy specimen from the left kidney tumor: large, abnormal multinucleated cells or spindle-shaped cancer cells were observed in a hematoxylin-eosin-stained tumor section, indicating clear cell renal carcinoma with sarcomatoid differentiation. **(B)** Postoperative specimen. **(C, D)** Postoperative pathological report: high-grade renal cell carcinoma with sarcomatoid components exceeding 50% and pronounced necrosis.

On 2 June 2022, a 40-year-old female patient was first admitted to our hospital, presenting with a persistent dry cough lasting 6 months and a 1-week history of shortness of breath. Physical examination revealed multiple palpable and enlarged left supraclavicular lymph nodes with indistinct borders, coalescing into a mass approximately 2.0 cm × 2.0 cm in size. Prior to admission, she was in good health with no personal or family history of diabetes, heart disease, or hypertension. She underwent contrast-enhanced computed tomography (CT) of the whole abdomen and chest. The abdominal CT revealed a 9-cm tumor in the left kidney (**Figure 1A1**), along with swollen retroperitoneal lymph nodes. Chest CT scan (**Figures 1A2–4**) demonstrated bilateral massive pleural effusion, multiple metastatic lesions in the pleura and lungs, and metastatic involvement of bilateral hilar, mediastinal, and cervical lymph nodes. Bone scintigraphy showed no evidence of metastatic involvement. Her creatinine level (49 umol/L) and glomerular filtration rate (GFR) (126 mL/min) were within normal range. To establish a definitive diagnosis, a CT-guided biopsy of the left renal tumor was performed. Histopathological examination indicated clear cell renal carcinoma with sarcomatoid differentiation. Immunohistochemistry revealed the following profile: Vimentin (+); CA9 (+); CD10 (+); Pax-8 (+); CKpan (+) (**Figure 2A**). Based on these findings, the clinical diagnosis was left mSRCC, clinical stage T3aN1M1.

Following a multidisciplinary consultation among the departments of oncology, radiology, and urology, urology specialists noted that the patient had a large tumor (cT3aN1M1) with multiple distant metastases. Her Eastern Cooperative Oncology Group (ECOG) performance status score was recorded as 3, indicating significant limitations in daily living activities. Under such circumstances, direct surgical resection was considered extremely risky, with uncertain long-term outcomes.

The KEYNOTE-426 clinical trial, presented at the 2021 ASCO meeting, broadened treatment options for metastatic renal cell carcinoma (mRCC). Pembrolizumab, an anti–PD–1 monoclonal antibody, and axitinib, a vascular endothelial growth factor receptor tyrosine kinase inhibitor VEGFR-TKI), demonstrated antitumor activity in patients with untreated mRCC. In accordance with KEYNOTE-426, we recommended first-line systemic treatment with a combination regimen of pembrolizumab plus axitinib, to which the patient consented.

On 18 June 2022, we initiated treatment with pembrolizumab (200 mg via intravenous infusion every 3 weeks) and axitinib (10 mg daily). To assess therapeutic efficacy, enhanced abdominal and chest CT scans were repeated every two cycles, in accordance with the Response Evaluation Criteria in Solid Tumors version 1.1 (RECIST 1.1). After two cycles of combined therapy with pembrolizumab and axitinib, a reduction in the size of both the primary tumor and metastatic lesions was observed. The patient experienced no discomfort and showed marked improvement in her dietary intake and overall quality of life. After four treatment cycles, all metastatic lesions had completely resolved (**Figures 1B1–4**).

Following 12 cycles of combination therapy, the patient reported moderate abdominal pain, nausea, and hematochezia. On 6 March 2023, a repeated CT scan demonstrated no significant change in the lesions; thus, we conducted gastrointestinal endoscopy. However, the endoscopy demonstrated no abnormality. Minor hemorrhagic complications are frequently observed in patients undergoing treatment with targeted therapies. Consequently, we discontinued axitinib administration in the 13th cycle, leading to a gradual alleviation of the patient’s symptoms. During cycles 13 to 22, the patient received pembrolizumab monotherapy. We repeated enhanced CT scans every two cycles to assess therapeutic effect and observed a stable disease status, with no signs of recurrence or metastasis. Our department conducted a collaborative consultation involving specialists from radiology and urology. Urology specialists suggested that the patient’s age, complete disappearance of all metastatic lesions, and the presence of an intact tumor capsule aligned with the indications for abdominal tumor resection.

Given the notable effectiveness of transarterial chemoembolization (TACE) in minimizing intraoperative bleeding and suppressing tumor activity, we devised a revised treatment strategy incorporating TACE. A total of 60 mg pirarubicin was administered through the trunk of the left renal artery, with two sessions of TACE completed during administration of pembrolizumab monotherapy. However, we failed to achieve the desired reduction in tumor size via TACE. An enhanced CT scan repeated on 20 September 2023 demonstrated that the therapeutic outcome remained stable (**Figures 1C1–4**).

Scheduled for surgical excision in October 2023, the patient had by then undergone immunotherapy for over a year. Throughout the treatment period, her levels of cortisol, aldosterone, adrenocorticotropic hormone, thyroid function, and other indicators remained within normal ranges.

**Figure 3:**
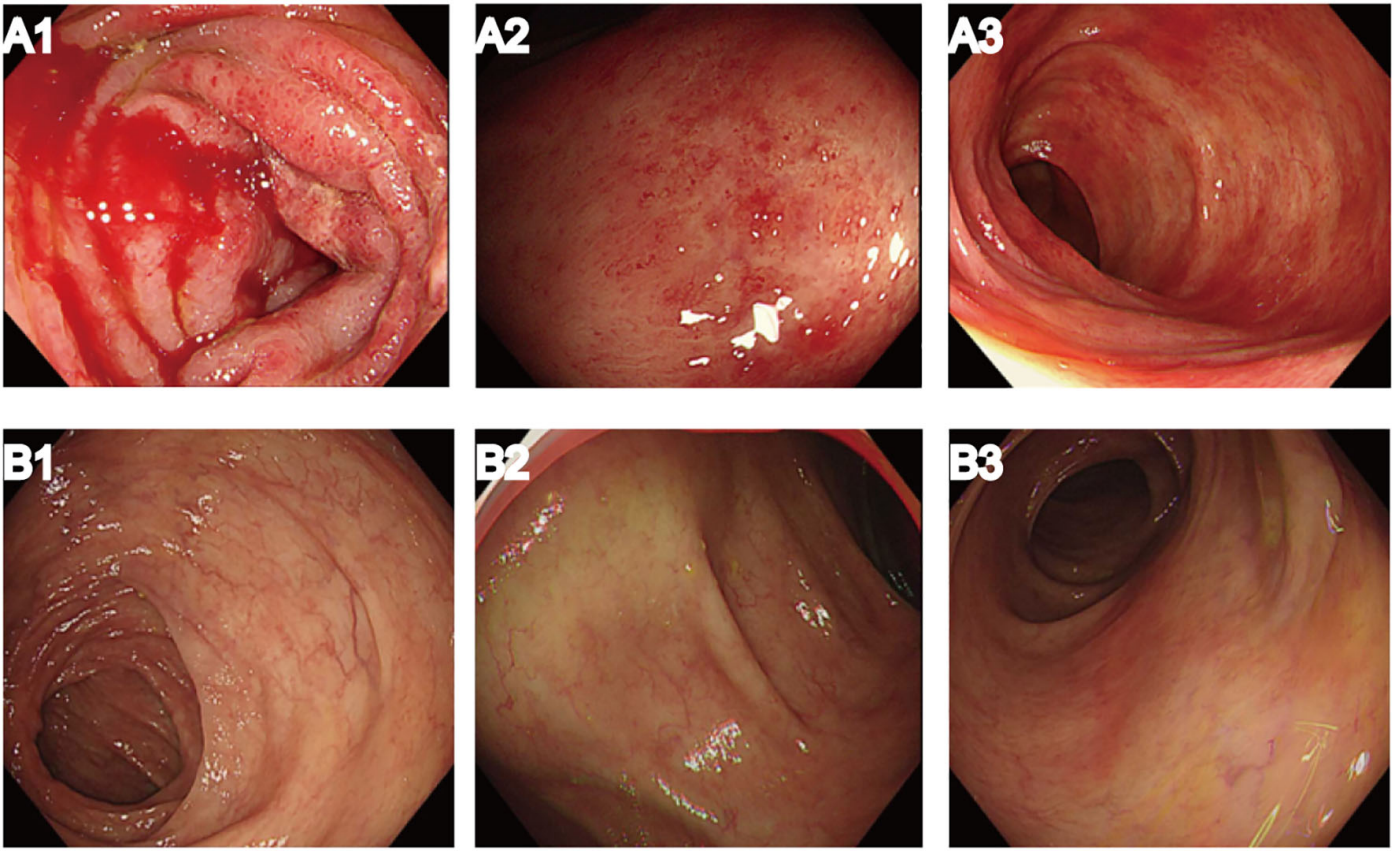
Results of painless colonoscopy before and after systemic corticosteroid treatment. **(A1–3)** Colonoscopy on 26 October 2023 demonstrated diffuse mucosal bleeding and edema. **(B1–3)** Colonoscopy on 20 March 2024, after systemic corticosteroid treatment, showed significant improvement in enteric erythema.

However, the patient was readmitted to our hospital due to abdominal pain accompanied by hematochezia, nausea, and vomiting. A repeat abdominal enhanced CT scan demonstrated edema and exudation in the ascending and transverse colons, suggesting the possibility of inflammatory bowel disease. On 26 October 2023, the patient underwent painless colonoscopy, which revealed diffuse mucosal bleeding and edema affecting approximately 10 cm of the transverse colon near the hepatic flexure (**Figure 3A1**). Endoscopy revealed widespread reddish spots, reminiscent of angiectasia within the enteric cavity (**Figures 3A2–3**).

Pathological examination demonstrated interstitial fibrosis and extensive edema, accompanied by capillary and venous dilatation in the submucosa extending into the mucosa. Upon further discussion within our department, it was deemed most probable that this complication arose as an adverse reaction to pembrolizumab, specifically suspected to be immune-mediated colitis. Thus, we discontinued pembrolizumab treatment and initiated therapy with systemic corticosteroid therapy. Additionally, the patient received symptomatic and supportive analgesics.

Twelve weeks after initiation of systemic corticosteroid treatment, the patient’s colitis symptoms gradually improved. After another 2 months, a repeat painless colonoscopy was performed on 20 March 2024, and significant improvement was seen in the enteric erythema (**Figures 3B1–3**). Due to the severity of the colitis, suspected to be pembrolizumab-induced immune-mediated colitis, immunotherapy was not restarted.

Every 3 months following the discontinuation of pembrolizumab, enhanced CT scans were conducted to assess the lesions. Fortunately, no changes were observed compared with the CT images captured on 20 September 2023.

**Figure 4:**
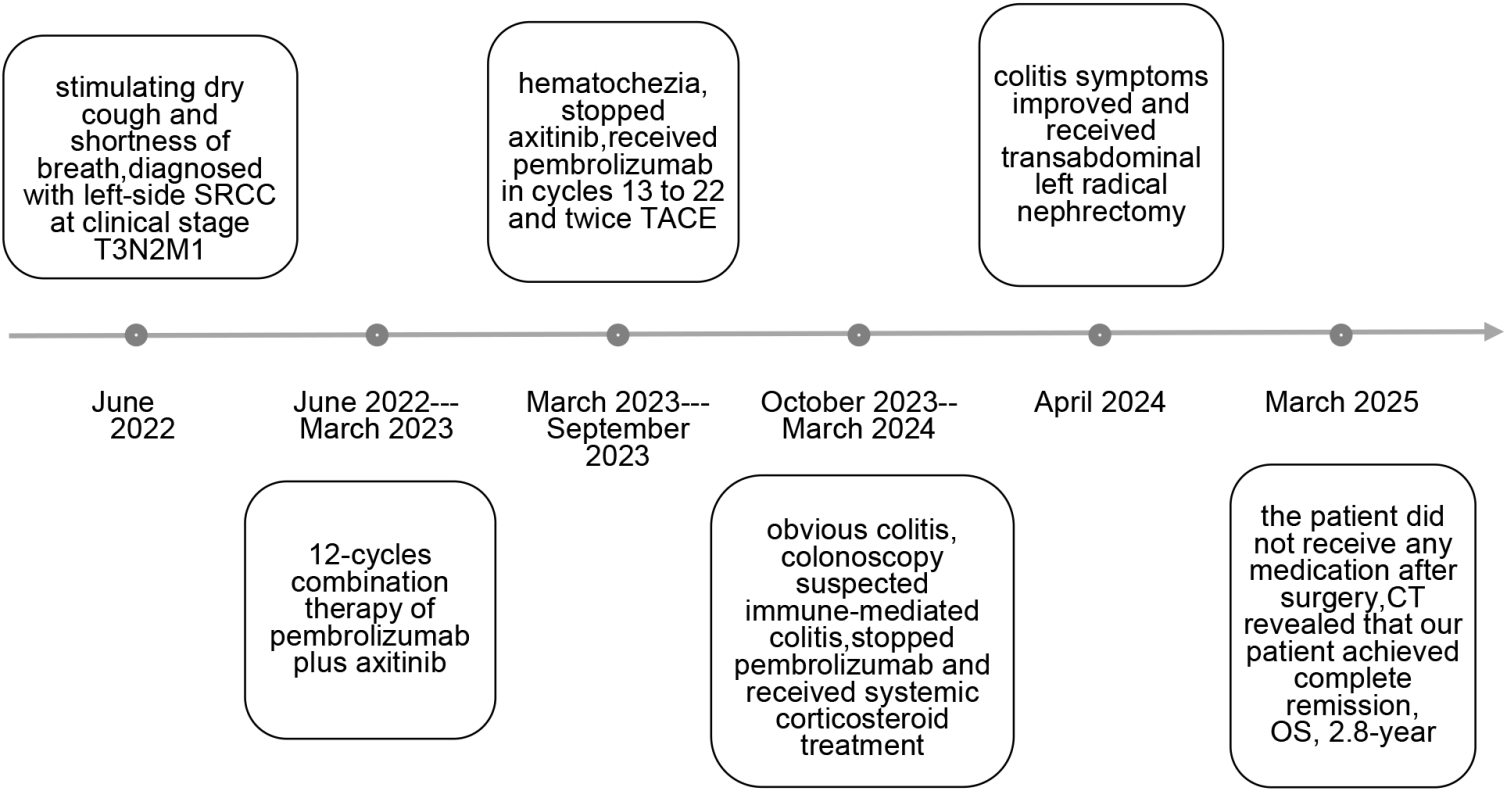
Timeline of clinical events.

On 2 April 2024, we performed a transabdominal left radical nephrectomy (**Figure 2B**). Although the tumor was sizeable, the prior treatments had a significant effect in reducing intraoperative bleeding and inhibiting tumor activity. The pathological diagnosis adopted was high-grade renal cell carcinoma with sarcomatoid components exceeding 50% and pronounced necrosis (**Figures 2C, D**). Immunohistochemistry revealed the following: PAX2 (+), PAX8 (+), CKpan (+), Vimentin: partial cells (+), CK7 (+), CD117 (-), TFE3: partial cells (+), ALK (-), P504S (-), HMB45 (-), MiTF (-), CA9 (-), CD10 (+), and Ki-67 (50%+). Following surgery, the patient experienced no apparent discomfort and did not receive any additional medication. Chest and abdominal enhanced CT scans during follow-up confirmed complete remission, consistent with RECIST 1.1 criteria. As of March 2025, her condition has remained stable (**Figures 1D1–4**), her overall health is good, and follow-up is ongoing (**Figure 4**).

## 3 Discussion

RCC is the most common kidney cancer, consisting of a variety of histologic subtypes (5). Sarcomatoid transformation in RCC is characterized by the conversion of epithelial neoplasm into malignant spindle cells, exhibiting an aggressive growth pattern and conferring a highly malignant phenotype (6). Some evidence indicates that the presence of a sarcomatoid component comprising 5% to 10% of the total tumor volume is associated with an increased risk of disease progression. This suggests that even a low level of sarcomatoid differentiation may hold clinical significance and should be documented in the pathological report (7–9). Sarcomatoid elements are present in approximately 5% of RCC; this percentage can increase to as much as 20% among patients with advanced stages of the disease (10). Generally, SRCC tumors are large and invasive, exhibiting metastatic potential in most cases (11). Therefore, mSRCC had a very poor prognosis in the pre-targeted therapy era. For instance, one described 63 cases of SRCC, including 47 cases of clear cell RCC and 16 of non-clear cell RCC. Another report described 204 cases of SRCC treated with nephrectomy, among which 168 patients died of the disease, with a mean survival of 1.7 years post-surgery (12). An increased percentage of sarcomatoid components in SRCC is indicative of a more adverse prognosis. When comparing patients with sarcomatoid differentiation ≥30% to those with <30%, a significant increase in mortality was observed in the former group (hazard ratio: 1.52; p = 0.018). The biological behavior of SRCC is not well understood, and its response to standard treatments is generally disappointing (13).

Currently, combination treatments such as ICIs plus TKI and ICI plus ICI regimens, have shown improved outcomes in mRCC. Among these combinations, an overall survival benefit has been demonstrated only for pembrolizumab plus axitinib and ipilimumab plus nivolumab. However, there are currently no prospective data comparing ICI–ICI combinations with ICI–VEGF inhibitor combinations. In the KEYNOTE-426 clinical trial, among patients with previously untreated mRCC, treatment with pembrolizumab plus axitinib resulted in significantly longer overall survival and progression-free survival, as well as a higher objective response rate, compared with sunitinib (14). Similar to numerous other tumors, RCC evades immune surveillance by upregulating programmed death-ligand 1 (PD-L1) expression. SRCC is typically a high-stage and high-grade tumor characterized by pronounced necrosis. Consequently, PD-L1 expression is anticipated to be higher in SRCC compared with RCC lacking sarcomatoid differentiation. Heightened PD-1 and PD-L1 expression in SRCC implies possible benefit from PD-1/PD-L1-targeted immunotherapy (15). In the era of targeted therapies for mRCC, increasing literature has explored their effectiveness in SRCC. With regard to molecular-targeted treatments for mRCC with sarcomatoid differentiation, a retrospective study involving 43 mSRCC patients reported that treatment with sorafenib, sunitinib, or bevacizumab achieved partial responses in a limited group of patients (19%) who had underlying clear cell histology and a >20% sarcomatoid component in the primary tumors. The median tumor shrinkage was 2%, median time to progression was 5.3 months, and median overall survival was 11.8 months. These results suggest that VEGF-targeted treatments have demonstrated limited benefits in mSRCC (16). However, recent advancements in molecular signatures hold promise for developing more effective treatment strategies. Axitinib is a potent and selective second-generation VEGFR-TKI with better safety and tolerability than sunitinib. It has been reported to achieve better disease control and symptom control when used to treat mRCC. Several recent case reports have demonstrated the efficacy of pembrolizumab plus axitinib in the treatment of mSRCC. Owing to the limited number of cases, these results should be interpreted with consideration of such limitations (17).

It has been reported that pembrolizumab plus axitinib can improve prognosis. In this case, the clinical outcome illustrates the effectiveness of combining pembrolizumab and axitinib for mSRCC. Unfortunately, evidence regarding the effectiveness of axitinib or pembrolizumab as monotherapy for mSRCC is currently lacking. However, given that VEGF—a target of the multi-tyrosine kinase inhibitor axitinib—is expressed at high levels in SRCC, and that PD-1 and PD-L1 expression is also elevated, pembrolizumab, an anti–PD–1 monoclonal antibody, may be beneficial through PD-1/PD-L1-targeted immunotherapy. Therefore, it is reasonable to treat mSRCC with a combination of TKI and ICI.

Limitations of this report include the small number of patients (single case) and the lack of detailed immunological characterization. On the other hand, this case suggests that pembrolizumab-induced immune-mediated colitis can be managed with steroid therapy, though the optimal dose and course of treatment require further investigation.

Although ICI or TKI therapies have expanded the therapeutic range for mSRCC and appear to achieve slightly better results than traditional treatments, systemic therapy remains challenging. Therefore, achieving localized curative surgical resection for mSRCC is of great importance. Although the efficacy of cytoreductive nephrectomy has been confirmed, many patients with locally advanced mSRCC/mRCC do not undergo surgical resection due to advanced disease stage, substantial tumor size, high risk of complications, or poor performance status (18). Careful patient selection and meticulous surgical techniques are essential in the treatment of patients with mRCC. These considerations should be further emphasized in the era of targeted therapies, as minor hemorrhagic complications are frequently observed in patients undergoing such treatments. Various studies have demonstrated that TACE can reduce tumor blood flow, accelerate tumor necrosis, shrink tumor volume prior to surgery, and minimize intraoperative blood transfusion (19). Obtaining more data on these approaches will help shed light on this important clinical issue. It is our hope that more patients with mSRCC will have the opportunity to undergo surgical resection and achieve better outcomes after receiving surgical resection.

## 4 Conclusions

Despite the sarcomatoid component constituting over 50% of the RCC in this case, we were able to maintain a relatively stable condition with no signs of recurrence or metastasis through a multidisciplinary treatment regimen. The female patient’s overall survival has reached 32 months, which is rare for mSRCC. Furthermore, the safety profile of the combination therapy regimen was manageable, and the patient’s tolerance and compliance were good. In this case report, the use of pembrolizumab plus axitinib demonstrated significant advantages and proved effective in promoting long-term survival. The findings from this case may help inform future treatment plans for mSRCC, and immunotherapy combined with targeted therapy could represent a promising treatment option for this disease (20).

## Data Availability

The original contributions presented in the study are included in the article/supplementary material. Further inquiries can be directed to the corresponding author.
